# Aortic dissection: visualisation of aortic blood flow and quantification of wall shear stress using time-resolved, 3D phase-contrast MRI

**DOI:** 10.1186/1532-429X-13-S1-P392

**Published:** 2011-02-02

**Authors:** Alex Pitcher, Tom E Cassar, Paul Leeson, Jane M Francis, Edward Blair, Paul B Wordsworth, J Colin Forfar, Steffen E Petersen, Michael Markl, Stefan Neubauer

**Affiliations:** 1 Oxford University, Oxford, UK; 2 University Hospital Freiburg, Freiburg, Germany

## Background

Aortic blood flow in healthy people is characterised by helical flow in the ascending and descending aorta, with corresponding patterns of wall shear stress (WSS) imposed upon the wall of the aorta by moving blood. Flow and WSS patterns in the aortic root and sinuses of Valsalva, and the flow patterns in diseased aortas are incompletely defined. We describe the use of a validated, time-resolved, 3D flow MRI sequence to characterize flow and WSS in the aorta, focusing on the aortic root, and demonstrate the flow and WSS in the aorta of healthy volunteers and patients with prior aortic dissection.

## Methods

**Figure 1:**
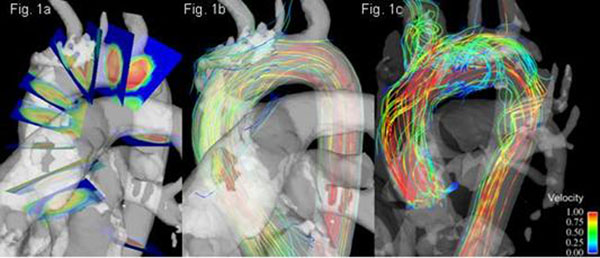
Figure 1 a. Orientation of planes for blood flow and wall sheer stress quantification. Fig 1b. 3D flow visualization of a 21 year-old healthy volunteer demonstrating non-helical flow at the aortic annulus, sinuses of Valsalva and sinotubular junction with helical flow in the distal ascending aorta, arch and proximal descending aorta. Fig 1c. 51 year-old male with Loeys-Dietz syndrome due to a novel mutation in the TGF β Receptor gene, with prior aortic valve and root replacement surgery, following Type A aortic dissection showing complex disturbance of flow with complex vortices and helices. Two distinct lumens are seen in the aortic arch, which reunite in the proximal descending aorta to form a pronounced right-handed helical flow jet.

10 healthy subjects and 3 patients with a prior history of aortic dissection underwent cardiovascular magnetic resonance imaging at 3T using a time-resolved 3-dimensional flow technique. Flow patterns were visualised as streamlines (Ensight, CEI). WSS quantification was undertaken at predefined aortic locations (Fig. 1a).

## Results

All healthy subjects showed retrograde systolic flow jets in the sinuses of Valsalva, with corresponding negative systolic mean axial wall shear stress (-0.22N/m^2^ ± SD 0.05) at this level compared to positive values at all other aortic levels (e.g. ascending aorta +0.54N/m^2^ ± SD 0.14, p<0.0005). All healthy subjects showed non-helical laminar flow in the aortic root, compared to right-hand helical flow typically seen in the ascending aorta and left-hand helical flow in the descending aorta (Fig. 1b). Correspondingly, circumferential wall shear stress was generally lower in the sinuses of Valsalva (-0.08 N/m^2^) compared to the distal ascending aorta (+0.17 N/m^2^), and mid-aortic arch (+0.22 N/m^2^). Prior repaired aortic dissection was associated with marked abnormalities of blood flow (Fig. 1c), with corresponding increases in axial WSS within the true lumen of the dissected aorta (typical axial WSS in the dissected ascending aorta was +0.9N/m^2^, compared to +0.54N/m^2^ in healthy controls).

## Discussion

We describe the flow and wall shear stress patterns in the aorta of healthy individuals and demonstrate the disturbances seen in patients with prior aortic dissection. Aortic dissection is associated with marked flow and shear stress abnormalities, which may predict long-term outcome after aortic dissection when evaluated in longitudinal studies.

